# Influence of Postharvest Natural Softening Conditions on the Nutritional Quality of Safou (*Dacryodes edulis* H.J.Lam) Flour: Rheological Properties of the Optimized Safou Flour

**DOI:** 10.1155/2024/6050991

**Published:** 2024-01-23

**Authors:** Alix Ntongme Mboukap, Mathilde Julie Klang, Bilkissou Njapndounke, Marie Madeleine Nanga Ndjang, Serge Cyrille Houketchang Ndomou, Hilaire Macaire Womeni

**Affiliations:** ^1^Research Unit of Biochemistry, Medicinal Plants, Food Sciences and Nutrition, Department of Biochemistry, Faculty of Science, University of Dschang, P.O. Box 67, Dschang, Cameroon; ^2^Laboratory of Microbiology, Department of Microbiology, Faculty of Science, University of Yaoundé I, Yaounde, P.O. Box 812, Cameroon; ^3^CRESA Forêt-Bois, Faculty of Agronomy and Agricultural Science, University of Dschang, P.O. Box 188, Yaounde, Cameroon

## Abstract

Safou (*Dacryodes edulis* H.J.Lam) is a seasonal fruit of great importance in the diet and economy of the populations of safou-producing countries. However, the valorization of this fruit is limited due to postharvest losses linked to the softening of the fruit, though this fruit is an essential source of macro- and micronutrients. Thus, this study is aimed at contributing to the valorization of softened safou by determining the optimum softening time, drying time, and temperature for good nutritional and rheological flour. The effects of the softening time, drying time, and drying temperature on the fat, protein, carbohydrate, dietary fibre, water, calcium, phosphorus, and potassium content were studied using the central composite design. The results showed that all the responses were affected by the factors. Also, the softening time of 4.75 days, drying time of 40.55 hours, and drying temperature of 41.83°C were the optimum conditions for producing safou flour with high nutritional value and good rheological properties. These optimum conditions resulted in safou flour with 58.95%, 12.40%, 22.40%, 4.38%, 4.91%, 2207.90 mg/1000 mg, 1898.30 mg/1000 mg, and 478.540 mg/1000 mg as lipid, protein, carbohydrates, fibre, moisture calcium, phosphorus, and potassium content, respectively. In addition, the pasting temperature, peak viscosity, breakdown, holding viscosity, final viscosity, setback, setback ratio, and stability ratio of the aforementioned flour were 50.42°C, 58 cp, 0.5 cp, 57.5 cp, 61.5 cp, 4 cp, 1.07, and 1, respectively. Based on its nutritional value and rheological properties, softened safou flour can be used in food formulation, thus contributing to the valorization of safou fruits.

## 1. Introduction

Over the last few years, there has been a focus on nonconventional crops with local and industrial development potential [1]. Rainforest species, such as *Dacryodes edulis*, *Irvingia gabonensis*, and *Baillonella toxisperma*, have attracted special attention. However, *D. edulis* is the most popular as it is cultivated in many countries of central Africa and the Gulf of Guinea [2]. Safou emerges as an economic crop in Cameroon because it is cultivated in many parts of the country and contributes to poverty reduction [3]. During the fruit-producing season, Cameroon exports safou to neighbouring countries and European countries such as Belgium, France, and Switzerland. Safou fruit, commonly known in Cameroon as “plum,” is ellipsoid drops, 4-8 cm long by 3-6 cm wide. The safou fruit ranges in size, and the skin and pulp colour varies with variety [4]. It can be used in food, pharmaceutical, and cosmetic industries [1]. In food industries, pulp flour can be used for fortifying bakery and pastry products [5]. Furthermore, this edible part contains 59% water, 32-44 g of lipids, 14-26 g of protein, 32-38 g of carbohydrates, fibre, and 4-10 g of ash [6]. Also, except tryptophan, safou pulp contains all the rest of amino acid [7]. In addition, the aforementioned fruit is a good source of minerals such as calcium, potassium, zinc, sodium, magnesium, manganese, and iron [2]. Nonetheless, the potential of this fruit is not fully exploited due to important postharvest losses resulting from fruit softening [8]. The highest postharvest losses are estimated at 50% in two to three days after harvest [9]. It is in this context that there is the recycling of softened safou into flour or oil for its valorisation [2]. The fact that softened safou pulps are still rich in nutrients, there is a need to understand their softening process and drying conditions. It is in this token that Dossou et al. [8] revealed that the softening process of safou pulp is influenced by the cultivar, picking method, mode of packaging, and temperature. In addition, Ndindeng et al. [10] highlighted that ripe and unripe fruits were most susceptible to deterioration due to the high level of polygalacturonase in the fruit. Also, Djedje et al. [1] found that the ripening phase (softening time) significantly influenced the firmness and the proximate components of safou pulp. Although the quality of fruit is affected by 1-methylcyclopropene during treatment, this compound increases the fruit's shelf life [11]. However, limited or no research has determined the optimum drying conditions for safou and the effects of softening time on the nutritional quality of softened fruit flour. Thus, the objective of this study was to determine the optimum drying conditions, softening time, and their effects on the nutritional value of safou flour and evaluate the rheological properties of the resulting product.

## 2. Materials and Methods

### 2.1. Materials

The plant material was matured safou fruit collected from a farm in the locality of Kekem in the Haut-Nkam division (West Region of Cameroon). This locality was retained based on previous work. The skins of the fruits collected were bluish and whitish, while the pulp colour was greenish [4]. The size of the fruit was large, and the pulp was thick. The fruits were harvested in October 2021 and transported in bags with holes to provide ventilation. All the chemicals and reagents used in this study were analytically graded.

### 2.2. Methods

#### 2.2.1. Determination of the Optimum Drying Conditions and Softening Time for the Production of Safou Flour

*(1) Experimental Design*. To determine the optimal conditions for the production of safou flour with high nutritional value, the response surface methodology was used. The design chosen for this work was the central composite design. The factors chosen were the softening time (*X*_1_), the drying time (*X*_2_), and the drying temperature (*X*_3_). These factors and their range of variation were selected based on previous work and the literature review, and Table 1 shows the experimental domain. The latter domains were used to generate the experimentation matrix (Table 2).

**Figure 1:**
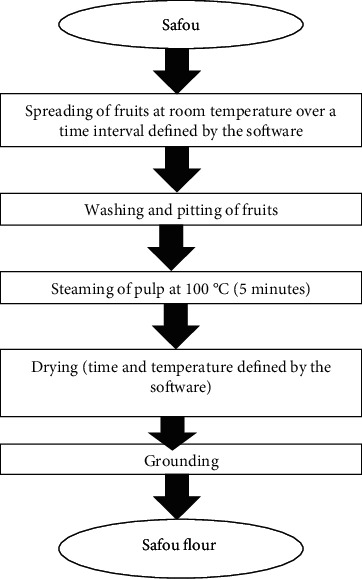
Procedure steps for the production of safou flour.

*(2) Production of Safou Flour*. The method proposed by Sika et al. [12] with slight modifications was used for safou flour preparation.  Figure 1 shows the production procedure used. Briefly, after collection, fruits were spread on bags at room temperature (20-22°C) over at different storage time as defined by the experimentation matrix (Table 2). About 10 fruits were randomly selected, washed, and pitted to separate the pulps from the pits, and then, the pulps were steamed for 5 minutes at a temperature of 100°C to reduce microbiological contamination. The pulps were spread on a rack and dried in an electric-ventilated oven (Venticell MMM Group, Sweden) at defined times and temperatures defined by the software (Table 2). The dried samples were ground using a dry blender (Moulinex, France), to obtain flour.

The latter was packaged in polystyrene bags and stored in a desiccator for further analysis.  Figure 1 shows the diagram of the production of softened safou flour.

*(3) Evaluation of Responses*.

- Determination of the Proteins, Lipids, Carbohydrates, Fibres, Ash, and Water Contents of the Safou Flour

The water content was determined by drying the samples in a Venticell brand ventilated oven at 105 ± 2°C until a constant weight was obtained [13]. Concerning the ash content, its determination was by incinerating the samples for 20 hours at 550°C. The nitrogen content was determined using the Kjeldahl method, and protein content was calculated according to the formula N^∗^6.25 [13]. When it came to the lipid content, it was evaluated according to the Soxhlet method [13]. In addition, the total carbohydrate content was determined by the differential method proposed by AOAC [13]. For the fibre content, the method described by Pauwels et al. [14] was used. All tests were done in duplicate.

(II) Determination of the K, P, and Ca Contents

The evaluation of these minerals was due to their abundance in safou pulp. Their determinations were done according to the method described by Pauwels et al. [14]. Precisely, 1 g of each safou powder sample was introduced into a porcelain crucible and calcined in a Carbolite Eurotherm muffle furnace at 450°C for 2 hours. After calcination, the ash was digested with 10 ml of nitric acid of 1 N concentration for 30 min then cooled and filtered using Whatman No. 4 filter paper into 50 ml volumetric flasks, and the volume was completed with distilled water to the mark. Finally, the sample was read directly using an atomic absorption spectrophotometer for K. When it came to the P assay, 2 ml of the ash extract was introduced into a test tube, and then, 6 ml of distilled water and 2 ml of nitro-vanada-molybdate reagent were added. The whole was homogenized and left to stand for 1 hour. Finally, the sample was assayed by spectrophotometry at 430 min. The absorbance obtained was projected onto the respective linear regression equations to determine the rate of Ca, K, and P in the safou powders.

*(4) Proposition of the Model*. The fitted response values were represented using the following second degree polynomial equation.

(1) $$\begin{array}{c} Y=b_{0}+ax_{1}+bx_{2}+cx_{3}+dx_{1}x_{2}+ex_{1}x_{3}+fx_{2}x_{3}+gx_{1}x_{1}+hx_{2}x_{2}+ix_{3}x_{3}+£, \end{array}$$

where *Y* is the expected response; *b*_0_ is the constant; *a*, *b*, and *c* are the linear coefficients; *g*, *h*, and *i* are the square coefficients; *d*, *e*, and *f* are the interaction coefficients; *x*_1_, *x*_2_, *x*_3_, *x*_1_*X*_2_, *x*_1_*x*_2_, *x*_1_*x*_3_, *x*_2_*X*_2_, *x*_2_*x*_3_, and *x*_3_*X*_3_ are the levels of the independent variables; *ε* is the error.

*(5) Validation of the Model*. To validate the model, the coefficient of determination (*R*^2^), absolute mean deviation analysis (AMDA), and the bias factor (Bf) were determined [15].

*(6) Multiple Optimization and Validation of the Optimum Conditions*. Multiple optimizations were done using all the responses, and the compromised conditions were determined. Validation of the compromised optimum condition was done based on desirability and the level of significance (*p* values <0.05) between the experimental and predicted values [15].

#### 2.2.2. Evaluation of the Rheological Properties of the Optimized Safou Powder

It should be highlighted that the safou flour obtained using the compromised optimum condition was called the optimized safou powder. The method described by Sanchez et al. [16] was used to determine the rheological parameters of the powder using the rapid velocity analyser (Perten RVA master). Precisely, in 25 ml of distilled water, 3 g of dry safou powder was dispersed, and different temperatures were applied. The particle size was not determined, and the moisture content was 4.91%. Holding viscosity was done at 50°C for 1 min, heating from 6°C.min^−1^ to 95°C, holding at 95°C for 5 min, and cooling at a rate of 12°C/min to 50°C, and final holding was done for 2 min at 50°C. The means of 2 replicates were calculated for each parameter. The pasting temperature (PT), peak viscosity (PV), holding viscosity (HV), and breakdown (BD) estimated from the ratio of PV and HV, final viscosity (FV), setback viscosity (SB) estimated from the ratio of FV and HV, the setback ratio (SBR = FV/HV), and stability ratio (STR = HV/PV) were evaluated.

### 2.3. Statistical Analysis

The experimentation matrix and the analysis of the optimization results were done using Minitab software version 18.0. A *p* value of 0.05 was considered significant. To plot response surface curves and isoresponse curves for multiple optimizations, SigmaPlot version 12.5 software was used. GraphPad Prism version 5 software was used to determine the significance threshold between the experimental and predicted values. This significance was done using the Student-Newman-Keuls comparison test at the 5% probability level.

## 3. Results and Discussion

### 3.1. Results

#### 3.1.1. Optimum Drying Conditions and Softening Time for the Production of Safou Powder

*(1) ANOVA and Coefficient of Determination for the Responses*.  Table 3 presents the experimental results obtained during the various tests as well as the adjusted values predicted by the software. From this, the lipid, protein, carbohydrates, fibre, moisture, calcium, phosphorus, and potassium contents vary, respectively, as follows: 55.01-65.50%, 7.97-12.21%, 17.95-28.26%, 4.25-4.99%, 3.56-5.40%, 409.4-2100.5 mg/1000 mg, 613.98-1535 mg/1000 mg, and 311.59-1110.20 mg.

Concerning ANOVA analysis, Table 4 presents the *p* values of the different factors for the responses. It appears that the linear effect and the quadratic effect of the drying temperature and the drying time significantly affect the lipid content of softened safou flour. The high value of the *R*^2^ of 83.64% shows that the linear and quadratic effects of the drying temperature contribute to 83.64% of the variation of the lipid content.

The protein content on the other hand was significantly affected (*p* < 0.05) by the linear effects of softening and drying times and the quadratic effects of these same factors. Also, the interaction between softening time, drying time, and drying temperature significantly affected the protein content. The *R*^2^ of 93.46% indicated that the linear and quadratic effects of the softening time and the drying time contribute to 93.46% of the variation of protein content.

Concerning the carbohydrate content, it was significantly influenced (*p* < 0.05) by the linear effects of the various factors and their quadratic effect. Here, the *R*^2^ showed that the linear effects of the various factors and their quadratic effect contribute to 97.16% of the variation in total carbohydrate content.

When it comes to the dietary fibre content, it was significantly affected (*p* < 0.05) by the interaction between drying time and drying temperature. The *R*^2^ of 80.94% revealed that the interaction between drying time and drying temperature contributed to variation in fibre.

Furthermore, the moisture content was significantly influenced (*p* < 0.05) by the linear effect of softening time and drying temperature. This was also affected by the quadratic effects of drying times and drying temperature and the interaction between drying time and drying temperature. The *R*^2^ showed that 92.92% of the variation in water content depended on the storage time and the drying temperature.

Also, the linear effects of drying time and drying temperature significantly affected (*p* < 0.05) the calcium content of softened safou powders. In addition, the interactions between storage time and drying time and between drying time and drying temperature as well as the quadratic effects of storage time and drying time significantly affect this parameter. These factors constitute a 99.33% (*R*^2^) variation in the calcium content.

For the phosphorus content of softened safou powders, the linear effects of storage time and drying temperature significantly affected (*p* < 0.05) it. Also, the interactions between the factors and the quadratic effects of factors significantly affected (*p* < 0.05) the potassium content. The *R*^2^ showed that 98.73% of the linear and quadratic effects contributed to the variation of phosphorus.

The linear effect of storage times and the interactions between the factors affected significantly (*p* < 0.05) the potassium content of the flour from softened safou. The *R*^2^ indicated that 91.16% of the linear effect of storage time and the interaction between drying time and drying temperature and storage time contributed to the variation of potassium.

*(2) Proposed Model and Validation*. The regression equations for each of the responses are given below ranging from (2)–(9). These equations showed the effect of each factor on the responses.

(2) $$\begin{array}{c} Y1=-132.1+2.71X1-0.800X_{2}+8.40X_{3}-0.012X_{1}X_{1}+0.00049X_{2}X_{2}-0.0900X_{3}X_{3}+0.0094X_{1}X_{2}-0.0325X_{1}X_{3}+0.0135X_{2}X_{3}, \end{array}$$

(3) $$\begin{array}{c} Y2=16.3-0.552X1+0.648X_{2}-0.636X_{3}-0.03938X_{1}X_{1}-0.003617X_{2}X_{2}+0.0065X_{3}X_{3}-0.01334X_{1}X_{2}+0.02947X_{1}X_{3}-0.00516X_{2}X_{3}, \end{array}$$

(4) $$\begin{array}{c} Y3=189.1-0.185X1+0.173X_{2}-7.175X_{3}+0.1292X_{1}X_{1}+0.00543X_{2}X_{2}+0.08170X_{3}X_{3}-0.00073X_{1}X_{2}-0.0304X_{1}X_{3}-0.01251X_{2}X_{3,} \end{array}$$

(5) $$\begin{array}{c} Y4=9.52+0.013X1-0.1889X_{2}-0.043X_{3}-0.00286X_{1}X_{1}-0.000175X_{2}X_{2}+0.01374X_{3}X_{3}-0.001424X_{1}X_{2}-0.00047X_{1}X_{3}+0.003885X_{2}X_{3,} \end{array}$$

(6) $$\begin{array}{c} Y5=-34.02-0.101X1+0.2907X_{2}+1.447X_{3}+0.00387X_{1}X_{1}-0.0001031X_{2}X_{2}-0.01374X_{3}X_{3}-0.00076X_{1}X_{2}+0.00017X_{1}X_{3}-0.00417X_{2}X_{3}, \end{array}$$

(7) $$\begin{array}{c} Y6=3972+316.9X1+84.7X_{2}-184.0X_{3}-10.324X_{1}X_{1}+1.885X_{2}X_{2}+0.529X_{3}X_{3}+2.083X_{1}X_{2}+3.01X_{1}X_{3}+1.532X_{2}X_{3}, \end{array}$$

(8) $$\begin{array}{c} Y7=13451-553.5X1+115.9X_{2}-474.4X_{3}-2.214X_{1}X_{1}-0.8934X_{2}X_{2}+4.163X_{3}X_{3}-0.653X_{1}X_{2}+11.218X_{1}X_{3}-0.973X_{2}X_{3,} \end{array}$$

(9) $$\begin{array}{c} Y8=4647-211.5X1-63.7X_{2}-95X_{3}+1.76X_{1}X_{1}-0.343X_{2}X_{2}+0.171X_{3}X_{3}+2.083X_{1}X_{2}+3.01X_{1}X_{3}+1.532X_{2}X_{3}, \end{array}$$

where *Y*_1_ − *Y*_8_ represent lipid, proteins, carbohydrates, fibre, moisture, calcium, phosphorus, and potassium content; *X*_1_ is the softening time (days); *X*_2_ is the drying time (h); *X*_3_ is the drying temperature (°C); and *X*_1_*X*_1_, *X*_1_*X*_2_, *X*_1_*X*_3_, *X*_2_*X*_2_, *X*_2_*X*_3_, and *X*_3_*X*_3_ are the levels of the independent variables.

Concerning the validation of the model, the AMDA and the bias factor values for each response were, respectively, equal to 0 and between 0.75 and 1.25 (Table 4). These obtained values imply valid models.

**Figure 2:**
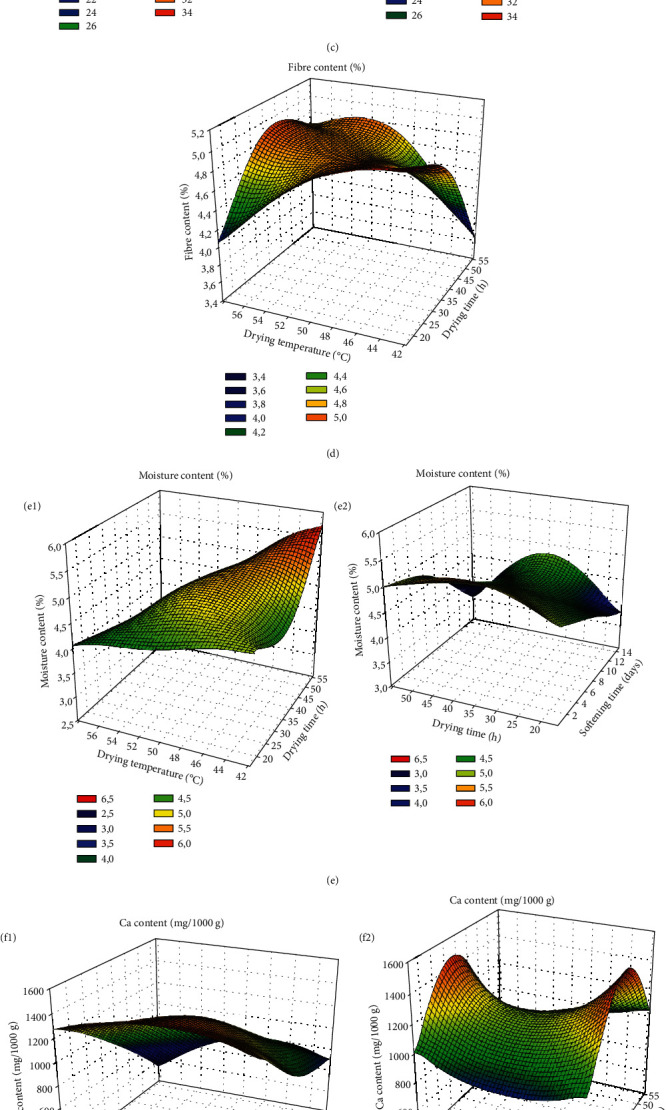
Response surface curves of lipid contents (a), proteins (b1, b2), carbohydrates (c1, c2), water (d1, d2), fibre (e), calcium (f1, f2), phosphorus (g1, g2), and potassium (h1, h2).

*(3) Response Surface Analysis of the Different Responses*. For response surface analysis, only the influencing factors on the responses were considered. The different response surface curves for each response are presented in Figure 2 (a-h2). It appears that the lipid content decreases with increasing drying temperature and softening time (a). As for the protein content (b1, b2), it decreases when the drying time is reduced and increases until it reaches a peak when the softening time increases and then begins to decrease. Regarding the carbohydrate content, it can be noted that the carbohydrate content increases with the drying temperature and decreases with the softening time (c1). For the fibre content, it can be seen from this figure that the fibre content increases with decreasing drying temperature and drying time (d). The moisture content (e1 and e2) decreases with increasing drying time and drying temperature, and increasing the softening time favours the increase in this content and then its decrease. The calcium content (f1 and f2) decreases when the drying time and the softening time are reduced, and the interaction between the drying time and the drying temperature allows the increase of this content rather than a decrease and an increase. As for phosphorus (g1 and g2), it increases when the softening time is reduced, and the drying temperature and the interaction of drying time and drying temperature favour an increase in this rate. When it comes to the potassium content (h1 and h2), it decreases when the softening time and the drying time are reduced and decrease with the interaction between the drying time and the drying temperature and then increase.

**Figure 3:**
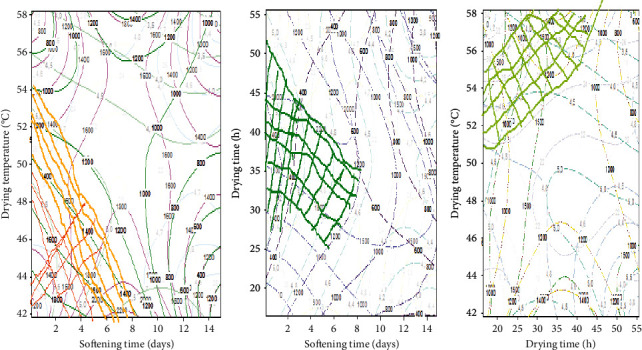
Overlaid contour plots of the lipid, protein, carbohydrate, fibre, moisture, calcium, phosphorus, and potassium contents for powder from softened safou fruit.

**Figure 4:**
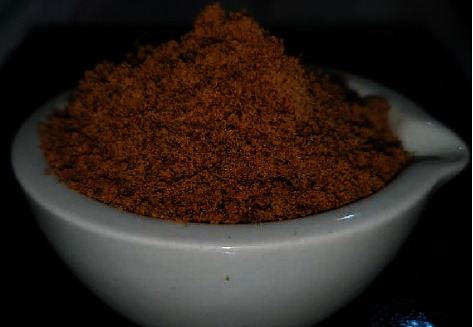
Optimized softening safou flour.

*(4) Multiresponse Optimization and Validation of the Compromised Optimum Condition*. Multiresponse optimization enables the building of an appropriate response surface model that groups all the responses and then tries to find a set of operating conditions for all responses by keeping them in the desired ranges.  Figure 3 shows the contour plot with the shaded zone representing the compromise optimum conditions for all the responses. From these zones, the values of the nutritional parameters in Table 5 were obtained. Also, from the latter table, the comparison of the experimental and predicted values can be seen. It should be noted that the desirability was closer to 1. Under this condition, the optimized softening safou flour (Figure 4) was obtained.

#### 3.1.2. Rheological Properties of the Optimized Safou Powder

**Figure 5:**
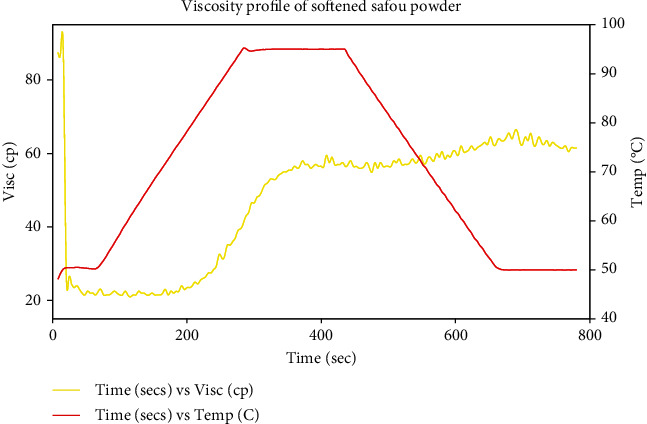
Viscosity profile of the optimum softened safou flour.

The pasting properties of the flour determine its suitability to be applied as a functional ingredient in food and other industrial products. Using the viscosity profile (Figure 5), the pasting parameters in Table 6 were obtained. It should be noted that the pasting temperature which is the minimum temperature at which there is a change in viscosity was 78.25°C. The peak viscosity was 58 cp, the holding viscosity was 57.5 cp, and the breakdown was 0.5 cp while the final viscosity was 61.5 cp. The setback viscosity was 4 cp, the setback ratio was 1.07, and the stability ratio was 1. The peak time was 324 sec.

### 3.2. Discussion

Lipids, proteins, and carbohydrates are the macronutrients of food and play essential roles in the body. They have structural, protective, and growth roles and good sources of energy for the body's cells [17]. These elements participate in the repair and maintenance of tissues. In the current study, different variations (increase and decrease) of the contents of lipids, proteins, and carbohydrates were observed. These different variations could be explained by the action of the different factors (softening time, drying time, and temperature). During the softening process, protein, lipid, and carbohydrate contents vary due to the action of enzymes that cause the reduction or increase of nutrients during softening. Indeed, during softening, there is initiation, propagation, and decline of the softening process. The propagation can cause modifications by increasing or decreasing lipid, protein, and carbohydrate contents. Djedje et al. [1] reported that during the propagation of softening, there is an increase in protein, ash, and moisture contents and a decrease in lipid, carbohydrate, and fibre contents. Also, the degradation of some carbohydrates can explain the change in the composition of the fruits during softening. In addition, the metabolism of polysaccharides in the cell wall and the starch hydrolysis during the softening process can result in the decrease of carbohydrates and some other components [18]. These changes during softening can be explained by the actions of enzymes contained in the fruit that have been activated during softening. According to Mamiro et al. [19], fruit softening is attributed to the reduction of insoluble pectin due to the action of some endogenous enzymes like cellulose pectinesterase and polygalacturonase leading to the increase or decrease in lipid, protein, and carbohydrates. Also, during softening, insoluble pectin in fruits degrades and modifies the constituents of the fruit [20]. Ndindeng et al. [10] reported that polygalacturonase activity in fruit pulp influenced fruit softening while Dossou et al. [8] reported that both the temperature and method of fruit storage influenced fruit softening and that very high temperatures favoured fruit softening. The drying time and temperature on the other hand influence the composition of the food since these nutrients are sensitive to them. During the drying process, there is nutrient content modification as the drying time and temperature influence the nutrient composition and contribute in some cases to their increase or decrease [21]. While drying, the moisture content of fruit decreases while protein, lipid, or other component of fruit probably increased. It can be explained by the fact that during the drying process, there is removal of water and condensation of other nutrients present in food. Depending on the time and the drying temperature, nutrient contents might increase or decrease due to the condensation or the deterioration of those nutrients [22]. Idah et al. [23] reported that the drying temperature and time affect the nutrient content of tomatoes. In addition, Miranda et al. [24] reported the same observation where increasing the drying time and temperature reduces the nutrient content of quinoa seeds. This shows that nutrients are sensible to drying time and temperature. Also, the increase or decrease in nutrients like lipids, proteins, and carbohydrates during the softening process can be explained by the action of microorganisms like bacteria especially Bacillus spp as shown by Mokemiabeka et al. [25]. These authors reported that *Bacillus subtilis* and *Bacillus safensis* degraded the pectin, amylose, and protein contents of safou pulp during softening. Bacillus spp can secrete pectinases, amylase, and proteases which are used in the degradation of safou pulp components during softening [25]. Also, Omogbai and Ojeaburu [26] showed that the development of microorganisms like bacteria (*Bacillus subtilis*) and fungi contributed to the softening and spoilage of safou fruits. When it comes to the coefficients of determination (*R*^2^), 83.64%, 93.46%, and 97.16%, respectively, for lipids, proteins, and carbohydrates greater than 75% show a valid model. According to Ross [27], when the coefficient of determination is greater than 75%, the model is valid. Also, the high coefficients of determination obtained being greater than 75% showed that the experimental values obtained are equivalent to the theoretical values predicted by the model [28]. For each response, the absolute mean deviation analysis (AMDA) was substantially equal to 0, and the bias factor was between 0.75 and 1.25. These conditions are similar to those recorded by Baranyi et al. [15]. The coefficients of determination, the AMDA, and the bias factor for each of the responses obtained in this study indicate that the model has been validated. The values obtained in the context of this work show that the use of safou flour or fruit can allow the human body to fulfil daily recommended allowances in these nutrients and consequently fight malnutrition in safou-producing countries. The results from this study are similar to those obtained by Silou et al. [29] in Congo-Brazaville on different sizes of safou fruit and superior to those obtained by Djedje et al. [1] in their study done on two varieties of fruit (*edulis* and *parvicarpa*) in Ivory Coast.

Minerals are essential micronutrients required for the normal functioning of the body. They are involved in several metabolic processes within the body [30]. Calcium is an essential constituent involved in the maintenance of skeletal growth and structure, muscular function, nerve conduction, and normal blood clotting; also, calcium is integral to multiple intracellular signalling pathways and serves as a cofactor in enzyme reactions. It is one of the most abundant minerals in the body, accounting for approximately 2% of adult body weight; 99% of body calcium is contained in bone, the rest being found in teeth, connective tissue, extracellular fluid, and blood (0.1%) [31]. It plays a role in insulin resistance and secretion [31]. The recommended calcium intake is between 300 and 1300 mg/day depending on the age, sex, and status of a human [32]. Phosphorus is present in all cells, acts as a component of bones and teeth, and a component of some lipids, and helps in the maintenance of pH, storage, and transfer of energy and nucleotide synthesis [33]. The recommended phosphorus intake is between 580 and 1055 mg/day [32]. Potassium is a micronutrient that is involved in the maintenance of fluid volume inside/outside of cells, and thus, normal cell function acts to blunt the rise in blood pressure in response to excess sodium intake and decreases markers of bone turnover and recurrence of kidney stones [33]. The recommended potassium intake is between 4500 and 5100 mg/day depending on the age, sex, and status of a human [33]. The decrease/increase observed in minerals in this study can be explained by the fact that during softening, there is a modification of fruit components. Djedje et al. [1] reported that during softening, there is an increase in ash content. Ash content indicates mineral elements in food. The increase or decrease in ash content during softening might explain the variation observed in mineral elements. Also, the variation observed can be explained by the presence of antinutrients like phytates, oxalates, tannins, and cyanides in safou pulp as demonstrated by Omogbai and Ojeaburu [26]. Concerning the validation of the model, according to [27], when the coefficient of determination is greater than 75%, the model is validated. In addition, when the coefficients of determination are greater than 75%, the experimental values obtained are equivalent to the theoretical values predicted by the model [28]. Based on the values of absolute mean deviation analysis (AMDA) (substantially equal to 0), the bias factor (between 0.75 and 1.25), and the coefficient of determination of more than 75% for each response, the model can be validated, and these conditions are in the same line with those obtained by Baranyi et al. [15]. The calcium, phosphorus, and potassium contents recorded in this study were higher than those obtained by Kadji et al. [2]. The calcium, phosphorus, and potassium values obtained during this work are within the range of recommended intake; thus, flour from softened safou is a good source of minerals for people in producing countries and can be used to supplement food in the populations of those countries.

Multiple optimizations build a suitable response surface curve model that groups all responses and then tries to find optimal conditions for all responses keeping them within desired ranges. It is a good approach for optimizing multiple responses because it uses the simultaneous optimization technique made popular by Derringer and Suich [34]. This procedure uses the desirability function [35]. The shaded region represents the compromise zone for all the responses. The obtained desirability closer to 1 indicates a satisfied optimal condition. The obtained nutrient content of the optimized flour is in the recommended range (lipids, proteins, and carbohydrates must provide 20-35%, 10-20%, and 50-55% of energy to the body, respectively). Also, the obtained softening day of 4.75 shows that the obtained optimized flour is free of heat-stable microorganisms such as Bacillus species [25]. Hence, this safou powder or fruit could be used in the human body to fulfil daily recommended allowances in these nutrients and therefore help to fight against malnutrition in developing countries. By so doing, it would help contribute to the valorization of local crops as shown by Njapndounke et al. [36] using “banane cochon” in the formulation of biscuits for diabetic persons.

Concerning the pasting properties, the lower values obtained in this study might be due to the higher lipid content and lower starch content of the flour from softened safou. Wani and Kumar [37] reported that higher fat contents have been shown to restrict starch from swelling as it absorbs water and inhibits interactions among starch molecules, as well as between starch and its paddles, and results in affecting pasting properties. It can also inhibit the directional arrangement of dispersed molecular chains of starch which induces the difficulty to retrograde. Safou powder is a great source of lipids, protein, fibre, ash, and carbohydrates. The interaction between safou powder components with starch or another ingredient in food formulation can help to ameliorate the texture and specific volume and even affect the organoleptic properties of products. Indeed, the higher the fibre content of an ingredient, the lower the specific volume of the cookies because the gas retention capacity is reduced [38]. As for the fat, it stabilizes and maintains the air bubbles incorporated during kneading. These make safou powder a suitable ingredient in the formulation of bakery products. De Lamo and Gomez [39] reported that oilseed flour modifies the rheological properties of dough with or without gluten. The values obtained in this work are lower than the ones obtained by [37] on chickpeas and dried green peas.

## 4. Conclusion

This study reveals that softened safou flour had proper rheological properties, and it is a good source of macronutrients and micronutrients. Also, the combination of the retained factors affects the nutritional value and rheological parameters of softened safou flour. In addition, the optimal conditions to obtain flour from softened safou with high nutritional value and rheological properties are as thus softening time (4.75 days), drying time (40.55 hours), and drying temperature (41.83°C). Hence, on the bases of the nutritional and rheological properties, softened safou flour (*Dacryodes edulis* H.J.Lam) can be used in fortifying food products intended for human consumption and, thus, used in the fight against malnutrition in safou-producing countries. The aforementioned points on safou flour would contribute to the reduction of postharvest losses of the safou fruit.

## Figures and Tables

**Table 1:** Experimental domain of factors.

Factors	Abbreviation	Range
Softening time (days)	*X*_1_	3-12
Drying time (h)	*X*_2_	24-48
Drying temperature (°C)	*X*_3_	45-55

**Table 2:** Experimentation matrix for the optimization process.

Trial number	*X*_1_ = softening time (days)	*X*_2_ = drying time (h)	Drying temperature = *X*_3_ (°C)
1	+1 (12.00)	-1 (24.00)	-1 (45.00)
2	0 (7.50)	0 (36.00)	0 (50.00)
3	+1 (12.00)	+1 (48.00)	-1 (45.00)
4	0 (7.50)	0 (36.00)	0 (36.00)
5	-1 (3.00)	-1 (24.00)	+1 (55.00)
6	+1 (12.00)	-1 (24.00)	+1 (55.00)
7	-1 (3.00)	+1 (48.00)	-1 (45.00)
8	+1 (12.00)	+1 (48.00)	+1 (55.00)
9	-1 (3.00)	-1 (24.00)	-1 (45.00)
10	0 (7.50)	0 (36.00)	0 (50.00)
11	0 (7.50)	0 (36.00)	0 (50.00)
12	-1 (3.00)	+1 (48.00)	+1 (55.00)
13	0 (7.50)	0 (36.00)	+*α* (58.17)
14	-*α* (0.15)	0 (36.00)	0 (50.00)
15	+*α* (14.84)	0 (36.00)	0 (50.00)
16	0 (7.50)	+*α* (55.60)	0 (50.00)
17	0 (7.50)	0 (36.00)	0 (50.00)
18	0 (7.50)	-*α* (16.40)	0 (50.00)
19	0 (7.50)	0 (36.00)	0 (50.00)
20	0 (7.50)	0 (36.00)	-*α* (41.84)

**Table 3:** Experimental and adjusted predicted values obtained from the different trials.

Trial order	*X*_1_ (softening time (day))	*X*_2_ (drying time (h))	*X*_3_ (drying temperature (°C))	Lipid content (%)		Protein content (%)		Carbohydrate content (%)		Fibre content (%)		Moisture content (%)		Calcium (mg/1000 g)		Phosphore (mg/1000 g)		Potassium (mg/1000 g)	
				Exp	Pre	Exp	Pre	Exp	Pre	Exp	Pre	Exp	Pre	Exp	Pre	Exp	Pre	Exp	Pre
1	+1 (12.00)	-1 (24.00)	-1 (45.00)	62.48	63.01	7.97	8.38	25.54	24.85	4.94	4.73	4.37	4.41	1680.10	1996.49	647.76	632.99	651.68	615.12
2	0 (7.50)	0 (36.00)	0 (50.00)	62.94	64.57	10.52	11.08	20.53	19.29	4.67	4.86	4.92	4.97	1900.50	1867.16	1134.77	1123.39	574.19	577.91
3	+1 (12.00)	+1 (48.00)	-1 (45.00)	62.33	61.96	8.40	8.27	24.26	24.68	4.25	4.80	4.98	4.89	1160.50	1185.34	613.98	633.28	733.91	748.97
4	0 (7.50)	0 (36.00)	0 (36.00)	64.22	64.57	10.54	11.08	19.22	19.29	4.99	4.86	5.13	4.97	1921.50	1867.16	1135.26	1123.39	575.41	577.91
5	-1 (3.00)	-1 (24.00)	+1 (55.00)	59.56	59.59	9.36	9.43	28.06	27.58	4.70	4.78	4.54	4.64	409.40	423.15	1027.08	1020.19	311.59	293.48
6	+1 (12.00)	-1 (24.00)	+1 (55.00)	55.01	56.36	10.72	10.85	28.26	28.17	4.30	4.47	4.14	4.16	1224.50	1241.14	1158.05	1164.98	433.41	569.74
7	-1 (3.00)	+1 (48.00)	-1 (45.00)	61.92	60.23	12.57	12.39	21.49	21.52	4.30	4.31	5.56	5.54	2100.50	2122.44	1632.96	1638.44	433.41	569.74
8	+1 (12.00)	+1 (48.00)	+1 (55.00)	57.14	58.54	9.42	9.51	25.43	24.99	4.88	5.09	3.37	3.64	1340.50	1357.55	881.20	931.66	433.41	294.04
9	-1 (3.00)	-1 (24.00)	-1 (45.00)	65.08	63.32	9.76	9.61	21.15	21.53	4.96	4.90	5.17	4.90	1028.50	1050.04	1535.90	1497.84	1110.20	1071.26
10	0 (7.50)	0 (36.00)	0 (50.00)	65.50	64.57	11.54	11.08	17.95	19.29	4.66	4.86	4.77	4.97	1900.50	1867.16	1137.92	1123.39	574.19	610.08
11	0 (7.50)	0 (36.00)	0 (50.00)	64.22	64.57	11.54	11.08	19.22	19.29	4.67	4.86	5.11	4.97	1900.50	1867.16	1135.26	1123.39	574.41	577.91
12	-1 (3.00)	+1 (48.00)	+1 (55.00)	60.61	59.73	11.44	10.97	23.94	24.56	4.63	4.66	4.32	4.28	2100.50	2123.10	900.00	927.18	311.59	345.10
13	0 (7.50)	0 (36.00)	+*α* (58.165)	55.85	54.52	12.21	12.28	27.93	28.14	4.89	4.95	3.56	3.34	1880.50	1855.93	1450.33	1408.66	651.68	581.17
14	-*α* (0.15)	0 (36.00)	0 (50.00)	56.11	58.58	9.98	10.39	25.90	25.54	4.70	4.72	5.40	5.54	1528.00	1497.43	1427.43	1440.85	311.59	364.09
15	+*α* (14.85)	0 (36.00)	0 (50.00)	59.31	57.36	8.53	8.19	28.16	28.61	4.75	4.85	4.78	4.62	1427.50	1400.15	770.39	738.36	1009.02	961.11
16	0 (7.50)	+*α* (55.60)	0 (50.00)	62.47	63.25	10.22	10.61	21.30	20.88	4.75	4.75	4.60	4.52	1802.50	1767.89	880.19	823.36	433.41	511.42
17	0 (7.50)	0 (36.00)	0 (50.00)	62.94	63.43	11.52	11.41	20.53	20.10	4.80	4.94	4.94	4.87	1920.50	2006.52	1124.77	1204.08	574.19	567.43
18	0 (7.50)	-*α* (16.40)	0 (50.00)	64.25	63.99	9.76	9.44	22.98	23.49	4.87	4.80	4.37	4.43	820.50	797.23	860.85	899.07	433.41	359.98
19	0 (7.50)	0 (36.00)	0 (50.000)	65.50	63.43	11.54	11.41	19.95	20.10	4.87	4.94	4.77	4.87	1918.50	2006.32	1227.92	1204.28	574.41	567.43
20	0 (7.50)	0 (36.00)	-*α* (41.84)	58.50	60.36	11.42	11.42	23.03	22.95	4.76	4.89	4.37	4.57	2260.50	227.19	2260.5	2227.19	501.43	576.52

Exp: experimental values; Pre: predicted values; *X*_1_: softening time; *X*_2_: drying time; *X*_3_: drying temperature.

**Table 4:** *p* values of the factors, coefficient of determination (*R*^2^), AMDA, and the bias factor of the different responses.

Sources	Lipid content	Protein content	Carbohydrate content	Fibre content	Moisture content	Calcium	Phosphore	Potassium
*X*_1_	0.505	0.001^∗^	0.002^∗^	0.808	0.001^∗^	0.090	0.001^∗^	0.001^∗^
*X*_2_	0.683	0.024^∗^	0.006^∗^	0.067	0.635	0.001^∗^	0.115	0.089
*X*_3_	0.009^∗^	0.074	0.001^∗^	0.605	0.001^∗^	0.001^∗^	0.008^∗^	0.955
*X*_1_ *X*_1_	0.004^∗^	0.000^∗^	0.001^∗^	0.158	0.217	0.001^∗^	0.010^∗^	0.179
*X*_2_ *X*_2_	0.900	0.033^∗^	0.007^∗^	0.052	0.033^∗^	0.001^∗^	0.001^∗^	0.074
*X*_3_ *X*_3_	0.002^∗^	0.248	0.001 ^∗^	0.583	0.001^∗^	0.424	0.001 ^∗^	0.865
*X*_1_ *X*_2_	0.483	0.002^∗^	0.894	0.146	0.600	0.001^∗^	0.071	0.006^∗^
*X*_1_ *X*_3_	0.322	0.004^∗^	0.042^∗^	0.831	0.962	0.063	0.001^∗^	0.059
*X*_2_ *X*_3_	0.275	0.102	0.029^∗^	0.001^∗^	0.009^∗^	0.001^∗^	0.008^∗^	0.017^∗^
*R* ^2^	83.64%	93.46%	97.16%	80.94%	92.92%	99.33%	98.73%	91.16%
AMDA	0.0	0.00	0.00	0.0	0.00	0.00	0.00	0.00
Bf	1.0002	1.0004	1.0003	1.0001	1.0005	1.0001	1.0005	1.0001

*X*_1_: softening time; *X*_2_: drying time; *X*_3_: drying temperature; *X*_1_*X*_1_: quadratic effect of softening time; *X*_2_*X*_2_: quadratic effect of drying temperature; *X*_3_*X*_3_: quadratic effect of drying temperature; *X*_1_*X*_2_: interaction between softening time and drying temperature; *X*_1_*X*_3_: interaction between softening time and drying temperature; *X*_2_*X*_3_: interaction between drying time and temperature; *R*^2^: coefficient of determination; AMDA: absolute mean deviation analysis; Bf: bias factor of the ^∗^factor with significant effect *p* < 0.05.

**Table 5:** Compromised optimum conditions and validation.

Compromised optimum conditions	Softening time (days)	Drying time (h)	Drying temperature (°C)
	4.75	40.55	41.83
Validation of optimal conditions			
*Responses*	Experimental values		Predicted values
Lipid content (%)	58.95 ± 0.28^a^		58.96^a^
Protein content (%)	12.40 ± 0.14^a^		12.44^a^
Carbohydrate content (%)	22.40 ± 0.2^a^		22.55^a^
Fibre content (%)	4.38 ± 0.15^a^		4.48^a^
Moisture content (%)	4.91 ± 0.29^a^		4.98^a^
Calcium (mg/1000 g)	2207.90 ± 0.19^a^		2208.81^a^
Phosphorus (mg/1000 g)	1898.30 ± 0.20^a^		1898.40^a^
Potassium (mg/1000 g)	478.540 ± 0.16^a^		478.556^a^
*Desirability*	0.92		

The values of the row with the same letter do not differ significantly (*p* > 0.05).

**Table 6:** Pasting parameters of the optimum softened safou powder.

Pasting parameters	Values
Pasting temperature	78.25 ± 0.03
Peak viscosity (PV)	58.00 ± 15.55
Breakdown (BD)	0.50 ± 0.71
Holding viscosity (HV)	57.50 ± 16.26
Final viscosity (FV)	61.50 ± 17.68
Setback (SB)	4.00 ± 1.41
Setback ratio (SBR = FV/HV)	1.07 ± 0.00
Stability ratio (STR = HV/PV)	1.00 ± 0.01
Peak time	324.00 ± 0.00

## Data Availability

All data generated or analyzed as part of this work are presented in this article.
